# Exemplifying the next generation of antibiotic susceptibility intensifiers of phytochemicals by LasR-mediated quorum sensing inhibition

**DOI:** 10.1038/s41598-021-01845-8

**Published:** 2021-11-17

**Authors:** Arpit Shukla, Gaurav Shukla, Paritosh Parmar, Baldev Patel, Dweipayan Goswami, Meenu Saraf

**Affiliations:** 1 Department of Microbiology & Biotechnology, University School of Sciences, Gujarat University, Ahmedabad, Gujarat 380009 India; 2 Department of Biological Sciences & Biotechnology, Indian Institute of Advanced Research, Gandhinagar, Gujarat 382426 India; 3 Pandit Deendayal Energy University, Raysan, Gandhinagar, Gujarat 382426 India

**Keywords:** Microbiology, Antimicrobials

## Abstract

There persists a constant threat from multidrug resistance being acquired by all human pathogens that challenges the well-being of humans. This phenomenon is predominantly led by *Pseudomonas aeruginosa* which is already resistant to the current generations of antibiotic by altering its metabolic pathways to survive. Specifically for this microbe the phenomenon of quorum sensing (QS) plays a crucial role in acquiring virulence and pathogenicity. QS is simply the cross talk between the bacterial community driven by signals that bind to receptors, enabling the entire bacterial microcosm to function as a single unit which has led to control *P. aeruginosa* cumbersome even in presence of antibiotics. Inhibition of QS can, therefore, be of a significant importance to curb such virulent and pathogenic strains of *P. aeruginosa*. Natural compounds are well known for their antimicrobial properties, of which, information on their mode of action is scarce. There can be many antimicrobial phytochemicals that act by hindering QS-pathways. The rationale of the current study is to identify such natural compounds that can inhibit QS in *P. aeruginosa* driven by LasR, PhzR, and RhlR dependent pathways. To achieve this rationale, in silico studies were first performed to identify such natural compounds which were then validated by in vitro experiments. Gingerol and Curcumin were identified as QS-antagonists (QSA) which could further suppress the production of biofilm, EPS, pyocyanin, and rhamnolipid along with improving the susceptibility to antibiotics.

## Introduction

The idea that microbes can evolve to a level where they become supremely resistant to noxious substances such as metals, radionuclides and more prudently, antibiotic came up with a superbug, *Pseudomonas putida* which acquired phenotypic traits that made this microbe impervious to multiple antibiotics^1–3^. Further, the unique trait of horizontal gene transfer in bacteria has led the spread of these multidrug resistance traits among different communities of bacteria due to which it is predicted that only a meagre amount of antibiotics will be functional while, the other antibiotics will become obsolete in half a millennium’s time. Unsurprisingly, another noxious and nuisance multidrug resistant pathogen which is responsible for causing multiple infections in humans also belongs to the genus of *Pseudomonas* i.e., *P. aeruginosa*^4^. For *P. aeruginosa* to induce pathogenicity, it produces biofilm, phenazine, pyocyanin, pyoverdin, and rhamnolipid governed by multiple QS-pathways which can serve as lucrative phenotypic targets where upon any quantitative reduction of their production can constitutively suppress the pathogenicity of this microbe^5^.

Briefly, the activation of QS is a cell density dependent event where each individual cell secretes set of signals that travel to the neighbouring cells and interact with their specific receptors which modifies the metabolic pattern in a way where the entire community behaves alike which contributes to the virulence of the pathogen. In Gram-negative bacteria like *P. aeruginosa*, a family of molecules known as *N*-acylated homoserine lactones (AHLs) serve as soluble signals released by the bacteria which can diffuse in the environment to interact with the adjacent microbes. The receptive microbe will acquire a signal which binds to its specific receptors broadly addressed as AHL-receptors (AHLr). In *P. aeruginosa*, there exist three major AHLr, (i) LasR, (ii) PhzR, and (iii) RhlR; that can bind with AHLs, (i) *N*-(3-oxododecanoyl)-l-homoserine lactone (OdDHL), (ii) *N*-hexanoyl-l-homoserine lactone (HHL), and (iii) *N*-butyryl-l-homoserine lactone (BHL) respectively. Activation of LasR helps the bacteria to produce exopolysaccharide (EPS) where all the members in the community concurrently produces EPS, whereby promoting the production of biofilm. This trait singly can enhance the potency of being virulent manifolds. Simply reducing the biofilm producing ability of *P. aeruginosa* can reduce its pathogenicity which is why inhibition of LasR is being looked upon as a lucrative target to develop its inhibitor^5^. The cascade of LasR expression triggers the activation of PhzR which produces toxins such as phenazine and pyocyanin, and RhlR which produces rhamnolipid which is known to behave like a biosurfactant and alters the surface tension by changing the viscosity of the medium.

Apart from these three AHLr, there exist other proteins such as RetS, LadS, LasI, RhlI, VqsR, PqsABCDEHR, RsmAYZ etc. which can influence the QS pathways^6^. The metabolic mesh in *P. aeruginosa* is so convoluted that assessing impact of each protein becomes a challenge which is why the most feasible aspect of QS-inhibition is possible only with LasR, PhzR, and RhlR as they have direct phenotypic impact on bacterial behaviour^7^. Till date there are reports where phytochemicals can inhibit QS pathways, however, several studies have incorporated the identity of only a handful of such phytochemicals. Computational advancements have allowed us, in recent times, to screen thousands of compounds and pick potentially important hits which can further be taken into consideration for in vitro validation. IMPPAT (Indian Medicinal Plants, Phytochemistry And Therapeutics (IMPPAT) is a curated database consisting of ~ 9500 phytochemicals from ~ 1700 Indian medicinal plants which we screened under current study to identify potential hits that can serve as QS-antagonist for *P. aeruginosa* making use of computational studies^8^. This served as the first objective of the study presented in this manuscript. For this, the computational studies dealt with include High Throughput Virtual Screening (HVTS) followed by E-pharmacophore based ligand mapping as second level of screening. From thereon, top scored hits were again filtered using Extra Precision (XP) docking and receptor-ligand binding free-energy change by MM-GBSA. At this stage, for each protein top ten potential compounds from the entire database were identified and recurring top two phytochemicals were then computationally validated by molecular dynamics (MD) simulations. The *in-silico* validation was subsequently tested by performing in vitro assays for their inhibitory effect on EPS, biofilm, phenazine, and rhamnolipid by *P. aeruginosa*^6^*.*

The use, overuse, and abuse of antibiotics has now reached a threshold which has accelerated development of multidrug resistant microbes and so, it is inevitable to find a strategy where even by using the existing antibiotics in a lower concentration we can achieve desired control of microbes. Therefore, the second objective of the current study after identifying the QSA is to test its efficacy as an antibiotic conjugant. This was done by assessing the modulations in minimum inhibitory concentrations (MIC) of antibiotic when bacteria were treated in combination with QSA. This helped us to predict how bacteria would behave in presence of antibiotic under two different scenarios, (i) QS not inhibited and (ii) QS inhibited. This helped us to know that inhibition of QS will weaken the bacteria thereby making them more susceptible to the antibiotic. This assessment will provide a new premise of utilizing a coordinated methodology of microbial control supported by QSA's in combination with antibiotics.

## Materials and methods

### Protein modelling of PhzR and RhlR and comparative assessment of LasR, PhzR and RhlR

Three AHLr’s under study namely LasR, RhlR and PhzR of *P. aeruginosa*, LasR is the only one that has been crystallized and it’s the 3D structure is available at protein databank (PDB). Protein (LasR) with the PDB ID 2UV0 is used for the entire study, this protein consists of homo-dimer, where the monomeric chain of this protein was used for the study. The 3D structure prediction of RhlR and PhzR was performed using Swiss-Model making used of available sequences from UniProt having entry ids Q51786 and P5429 respectively. Swiss-Model made use of template protein 4Y15.2.A (PDB ID) to build model for PhzR, while protein 4Y15.2.B (PDB ID) to build model for RhlR, which are two different chains of same protein 4Y15 of PDB. Swiss-Model chose these two template proteins based on the best hits with maximum protein sequence percent identity to that with input query sequence. Once the models were built the quality assessments of models was performed making use of QMEAN and QMEANDisCo scores^9,10^ and Ramachandran plot assement was performed using MolProbity v4.4^11^. Lastly, the overall reliability and secondary quality check of the protein was performed using ERRAT analysis^12^. All the three proteins being related to AHLr’s their internal sequence similarity was performed using multiple sequence alignment which identified the identical conserved regions amongst these proteins, while structural similarity was seen by superimposing all the three proteins based in sequence homology at identical spatial co-ordinates using MatchMaker tool of UCSF Chimera v1.14. The co-ordinates of AHL binding site on all the three AHLr’s under current study were determined using CASTp 3.0 server (Computed Atlas of Surface Topography of proteins)^13^ which determined the co-ordinates of active site, volume of active site cavity, surface area of active site cavity and also provided the amino-acids that constituted the active site of all three proteins.

### Preparation of ligand library and library energy minimization

Each molecule from IMPPAT library was manually downloaded from PubChem in the SDF format with the CID provided by IMPPAT. After successful download of 9500 molecules, all these molecules were imported to Schrödinger Maestro for ligand preparation following to which they were used in ligand-based and receptor-based screening. Ligand preparation helps in generating the low energy structures and allow the option to expand each input’s structure according to its desired stereochemistry by generating variations on ionisation state tautomer’s ad ring confirmations. LigPrep wizard in Schrödinger Maestro was used to generate ionization states for each ligand structure with Epik^14,15^ at a physiological pH of 7.2 ± 0.2 unit. Rest other options were kept as default and the ligands were minimized by OPLS-2005 force field. The output files prepared on ligand minimization was used for High-Throughput Virtual Screening (HTVS) docking, E-pharmacophore feature mapping-based screening, Standard Precision (SP) docking, Extra Precision (XP) docking in Schrödinger Maestro.

### Protein preparation

For E-pharmacophore feature mapping-based screening and for docking, each protein under study was imported to Schrödinger Maestro and was prepared in “Protein preparation” wizard of Maestro. Here the protein was first pre-processed by adding hydrogens, converting selenomethionine to methionine and het states were generated by Epik for pH 7.0. In the next step of protein preparation, H-bond assignment was done using PROPKA for pH 7.0 for optimizing the protein. Once the protein was optimized, the restrained minimization of protein was done using OPLS-2005 force field^16–18^. These tasks were all performed using the Protein Preparation Wizard of Schrödinger Maestro^19,20^. This process was done individually done for each protein under study viz., LasR, PhzR and RhlR making new project for each protein individually in Schrödinger Maestro.

### Screening of QSA for LasR, PhzR and RhlR using HVTS, E-pharmacophore feature mapping-based ligand screening, SP, XP and MM-GBSA

**Figure 1:**
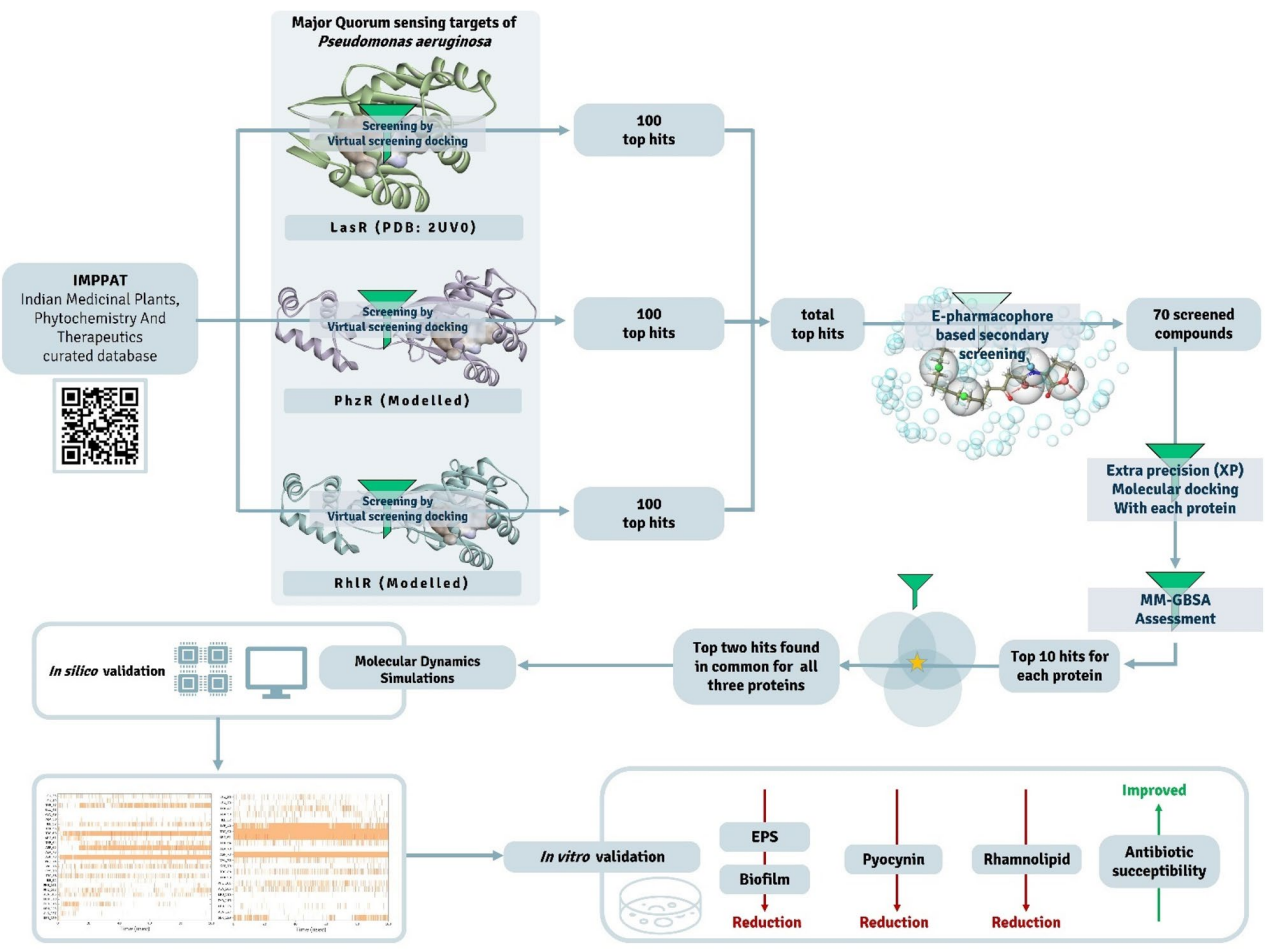
The workflow of the research presented in this article.

Ligands from IMPPAT were screened at four levels, (i) first the HVTS docking assessment was used to identify 100 top hits from 9500 molecules each protein, this was performed for all the three proteins individually, (ii) the total aggerate of top hits combining all the hits for all protein so obtained from HVTS docking were then subjected to E-pharmacophore feature mapping-based ligand screening for each protein. This further narrowed the pool of top hits to a total of 69 compounds including native ligands of all AHLr under study, (iii) These 69 compounds then subjected to two successive docking assessments of SP and XP, which identified top 10 hits for each of LasR, RhlR and PhzR, then (iv) lastly, receptor-ligand binding free-energy change by MM-GBSA for these top 10 compounds was performed to rank the compounds based on the degree of predicted spontaneity at which they interact with these proteins. Common recurring top two molecules having ability to interact with all three proteins LasR, RhlR and PhzR were identified, which were then subject to MD simulations. The workflow for the same is represented in Fig. 1.

For HVTS, SP and XP docking assessments, the optimized and minimized protein from the previous step was used. The grid at the exact same co-ordinates as that of the cavity predicted by CASTp 3.0 was prepared with the box of the size 10 Å × 10 Å × 10 Å was prepared using ‘Receptor grid generation’ feature of Glide module in Schrödinger Maestro. The output file of (i) receptor grid generation and (2) prepared minimized ligands were imported in the ‘Ligand docking’ window of Glide module in Schrödinger Maestro. Under the settings, the precision of docking was set as HVTS, SP or XP based on the stage of screening procedure where this is performed, ligand sampling was set as ‘Flexible’ and the Epik state penalties were added to docking score. The output was set to show only the best pose. The entire docking was performed using Glide module in Schrödinger Maestro^19,20^.

The E-Pharmacophore method was employed to achieve the advantages of both ligand- and structure-based approaches of generating energetically optimized, structure-based pharmacophores to rapidly screen phytochemicals. The E-pharmacophore models for (i) LasR-ODDHL, (ii) PhzR-HHL, and (iii) RhlR-BHL were imported simultaneously into Maestro workspace and the E-pharmacophore hypothesis were developed through the ‘Receptor-ligand complex’ of ‘Develop Pharmacophore model’ wizard in Phase module of Schrödinger Maestro. All 7 possible features were included in developing the hypothesis. Each hypothesis was used to screen the ligand data set and for this, ‘Ligand Based Screening’ wizard of Phase module was used.

Molecular mechanics generalized Born surface area (MM-GBSA) calculation was used to calculate the binding free energy change^21–23^ using the Prime wizard of Schrödinger Maestro. Binding energy for each receptor-ligand complex was determined by using OPLS-2005 force field. Equation employed for free energy calculation is as follows:

1 $$\Delta {\text{GBind}} = \Delta {\text{EMM}} + \Delta {\text{GSolv}} + \Delta {\text{GSA}}$$

Here, ΔEMM represents the variation between the minimized energy of the receptor–ligand complexes; ΔGSolv represents the variation between the GBSA solvation energy of the receptor–ligand complexes and the sum of the solvation energies for the protein and ligand. ΔGSA contains some of the surface area energies in the protein and ligand and the difference in the surface area energies for the complexes.

### Molecular dynamics (MD) for simulation studies

Protein–ligand complexes possess dynamic characteristics and therefore analysing their movements at the atomistic level utilising MD simulation becomes essential in understanding the key physicochemical phenomena. Desmond (Schrödinger Release 2018-4) was used to perform simulation of all the AHLrs in the presence of the top lead phytochemical. To ensure the accuracy of results of MD simulation, LasR, PhzR and RhlR complex with their native ligand were used as a reference set. Docked complexes were prepared for MD using Protein Preparation Wizard of Schrödinger Maestro to ensure of pre-simulation protein relaxation. Briefly, the parameters for simulation involved a solvent model: TIP3P; with orthorhombic box with buffer space around the periphery of protein to be 13 Å. Neutralisation was performed with the placement of Na + ions and a salt concentration of 0.15 M Na^+^ and Cl^−^ counter ions to simulate the background salt at physiological conditions. Steepest descent energy minimization was performed, and the simulation was proceeded for 100 ns with NPT (constant Number of particles, Pressure, and Temperature) with 300 K and 1.01 bar, constant volume, smooth Particle-Mesh-Ewald (PME) technique. For the simulation time of 100 ns, the energy recording interval was set at 1.2 ps and simulation trajectories recording interval was set at every 9.6 ps for each of the docked complexes. On completion of simulation, Simulation Interaction Diagram Wizard of Desmond package was used to evaluate the trajectories for Root Mean Square Deviation (RMSD), and ligand–protein contact profiles, and ligand–protein contact types and timeline.

### In vitro assessment on growth, EPS, biofilm, pyocyanin, rhamnolipid and antibiotic susceptibility on *P. aeruginosa*

The influence of 6-Gingerol and Curcumin on the growth kinetics of *P. aeruginosa* was estimated by plotting the optical density of the cell concentration against time. For this, activated culture of previously isolated *P. aeruginosa* AM26 (GenBank accession no.: MN871520) was used to inoculate different sets of nutrient media, (i) control flask without any supplementation of either 6-Gingerol or Curcumin, (ii) individual flasks supplemented with 10, 20, 30, 40, and 50 μg/mL concentration of 6-Gingerol (Sigma-Aldrich G1046) and 15, 30, 45, 60, and 75 µg/mL concentration of Curcumin (HiMedia, RM1449)^4,5^. All the sets were inoculated with OD 0.6 of activated culture and incubated on orbital shaker at ~ 150 rpm for aeration and agitation and for spectrophotometry, aliquots were withdrawn at every 60’ interval till the culture had entered its stationary phase of growth. The concentration of QSA at which there was significant deviation of growth of the bacterium as compared to the control flask was considered as the minimum threshold concentration (minimum inhibitory concentration, MIC) of the QSA as beyond this, the growth kinetics of *P. aeruginosa* was severely impeded and subsequently its growth was also inhibited considerably. The determination of MIC of QSA compounds helped in designing of experiments pertaining to check any modulation in antibiotic susceptibility by *P. aeruginosa* AM26.

After establishing the antimicrobial efficacy of 6-Gingerol and Curcumin, their QSA traits were determined for the inhibition of EPS, biofilm, pyocyanin and rhamnolipid production. The EPS production was carried out in the EPS medium supplemented with varying concentrations of 6-Gingerol and Curcumin as discussed by Shukla and colleagues^24–26^. The reduction in EPS production was gravimetrically quantified and represented in terms of g%(w/v) while, for the biofilm assay, nutrient broth was supplemented with 3% sucrose sugar and the culture was allowed to grow for 24 h under static condition whereafter, the biofilm was quantified spectrophotometrically as per the protocol discussed by O’Toole^27^. The concentration of 6-Gingerol and Curcumin for both the assays were same as that of the growth kinetic study while the control set did not contain any trace of these QSAs.

For reduction in pyocyanin or pigment production of *P. aeruginosa* AM26, the culture was allowed to grow in nutrient medium with varying concentrations of QSA, as mentioned above, and incubating the flask at 37 ± 4 °C under shaking conditions overnight. The next day, trichloroacetic acid was added to the culture medium and to it 5 mL supernatant, 3 mL of 100% chloroform was added and consequently, pyocyanin was recovered by adding 1 mL of 0.2 N HCl to this mixture. Pyocyanin was estimated spectrophotometrically at 520 nm and to determine its correlation with the biomass, which was also optically determined at 600 nm, the final pyocyanin activity inhibition is represented as a ratio of the pyocyanin to the biomass (OD 520/600)^7^.

The inhibitory effect of QSA in reduction of rhamnolipid production was quantified by extracting rhamnolipid from an overnight grown culture. To 500 µL of cell supernatant, 1 mL of 100% diethyl ether was added and the other component was dried to evaporation following which, the sample was eluted in 500 µL of deionized water. 900 µL of Orcinal solution (0.19% w/v in 53% H_2_SO_4_) was then added to 100 µL of the eluent and subsequently, the mixture was heated (almost boiled) for half-an-hour and then allowed to cool at ambient temperatures for 15 °C. The rhamnolipid content was also spectrophotometrically quantified by measuring the OD of the sample at 421 nm and the final rhamnolipid activity is represented as the ratio of rhamnolipid production to the biomass (OD 421/600)^7^.

Pseudomonad infections are widely treated with ciprofloxacin (aminoglycoside) and ceftazidime (cephalosporin) antibiotics and therefore, to test the efficacy of the screened compounds, 6-Gingerol and Curcumin against these antibiotics, on the growth of *P. aeruginosa* was performed. The antimicrobial assay of 6-Gingerol and Curcumin was carried out against a previously reported *P. aeruginosa* AM26 and their inhibitory traits were contrasted with sub-minimum inhibitory concentrations of ciprofloxacin (HiMedia CMS1891) and ceftazidime (Sigma-Aldrich C3809). Briefly, the nutrient medium was supplemented with different 0.0 to 2.5 μg/mL concentration of the antibiotics, separately, with the difference in concentration of the antibiotics between each set of 0.5 μg/mL. The activated culture with an optical density (OD) of 0.6 was inoculated into the nutrient medium supplemented with the antibiotic and the culture was allowed to grow for 24 h on shaking conditions where the inhibition was deduced spectrophotometrically by determining the reduction in the OD of the culture at 600 nm^28^. Here the increase in the antibiotic susceptibility seen is significant or not was determined using Student T-test.

## Results

### Protein modelling of PhzR and RhlR and comparative assessment of LasR, PhzR and RhlR

Swiss-Model was used to prepare the 3D protein models of PhzR and RhlR, while the structure of LasR being available on PDB (ID: 2UV0), it was simply retrieved and used for further studies. For the 3D model of PhzR, QMEAN score was − 3.6 and Global Model Quality Estimation (GMQE) score was 0.75. MolProbity v4.4 performed the Ramachandran plot analysis, where it was found that 94.89% of residues of model were in the allowed region while, the 1.01% residues were rotamers and these were Val52 and Asn221. Pro95, Pro125, Ser117 and Ala94 were situated in the plot as Ramachandran outliers constituting a total of 2.13%. None out of 3870 bonds found in the model showed discordant bond in MolProbity analysis. Similarly, for RhlR, QMEAN score was − 2.04 and GMQE score was 0.77. 96.58% of residues of model were in the allowed region of Ramachandran plot while, Leu130, Pro20 and Asn218 constituted 1.46% rotamer outliers. Pro123 and Ile143 constituting 0.85% Ramachandran outliers. Gly06 showed discordant bond in MolProbity analysis out of 3894 bonds found in model. The cumulative MolProbity score for PhzR was 1.68 and for RhlR this score was 1.61.

**Figure 2:**
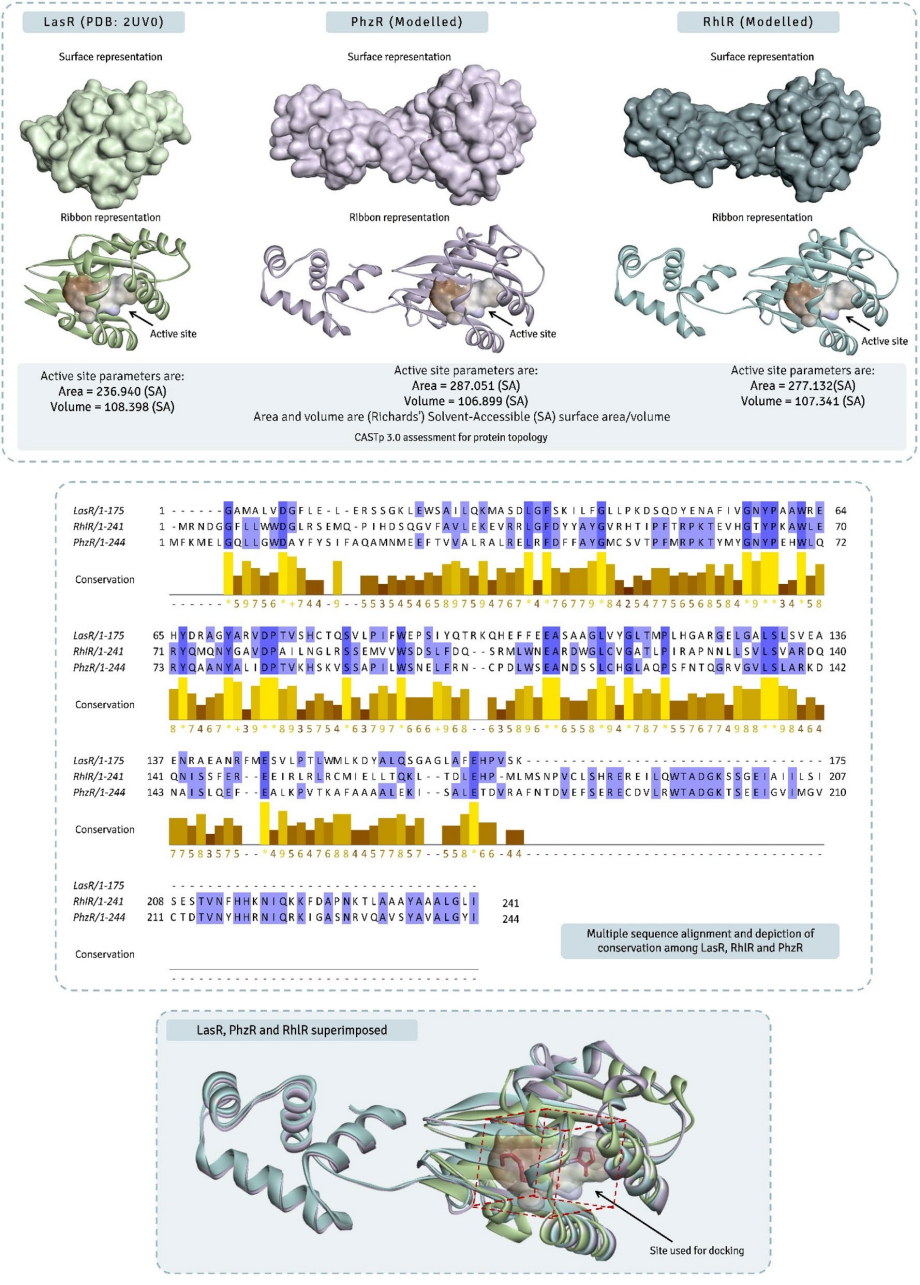
Structural and sequence comparison of LasR, PhzR and RhlR, further superimposition of all three proteins and identifying their accurate active site.

Structural comparison of LasR, PhzR and RhlR, suggested LasR to lack helix-turn-helix (HTH) domain, while both PhzR and RhlR possessed HTH domain. Multiple sequence alignment of the protein sequences of LasR, PhzR and RhlR suggested that there are several patches of sequences across the length which showed conservedness (Fig. 2). Percent identity assessment showed that LasR to have only ~ 19% similarity with PhzR and RhlR, while PhzR and RhlR were internally ~ 40% similar, despite this, when all the three proteins were superimposed, they show to have identical structure with similar active site for AHL as ligand, consisting of a five-stranded antiparallel β-sheet packed against three α-helices on each side. The cavity was found to be formed by a cluster of hydrophobic and aromatic residues as predicted by CASTp 3.0 protein topology assessment. The list of amino acids for each protein forming active site is represented in Table 1. Further, CASTp 3.0 assessment showed active site of all the three proteins having identical surface area and cavity volume (Fig. 2). This proposes that each of the three proteins may have diverse genealogy, but they share striking indistinguishable structural and functional activity.

**Table 1:** Amino acids at Active site prediction by CASTp.

Number	Amino acid	Number	Amino acid	Number	Amino acid
LasR (PDB: 2UV0)		PhzR		RhlR	
36	Leu	39	Ala	39	Ala
38	Gly	40	Tyr	40	Tyr
39	Leu	41	Gly	41	Gly
40	Leu	42	Met	43	Arg
47	Tyr	43	Cys	49	Thr
48	Glu	53	Thr	50	Arg
50	Ala	55	Met	51	Pro
52	Ile	56	Tyr	53	Thr
56	Tyr	57	Gly	54	Glu
60	Trp	59	Tyr	55	Val
61	Arg	63	Trp	56	His
64	Tyr	64	Leu	57	Gly
65	Asp	67	Tyr	59	Tyr
68	Gly	73	Ala	63	Trp
69	Tyr	76	Asp	64	Leu
70	Ala	78	Thr	67	Tyr
73	Asp	79	Val	68	Gln
75	Thr	91	Trp	71	Asn
76	Val	96	Phe	73	Gly
79	Cys	102	Leu	74	Ala
80	Thr	103	Trp	76	Asp
88	Trp	106	Ala	78	Ala
93	Tyr	111	Leu	79	Ile
101	Phe	126	Val	91	Trp
105	Ala	128	Val	96	Phe
110	Leu	130	Ser	102	Leu
				103	TRP
				106	ALA
				111	LEU
				128	VAL
				130	SER

### Screening of QSA for LasR, PhzR and RhlR using HVTS, E-pharmacophore feature mapping-based ligand screening

**Figure 3:**
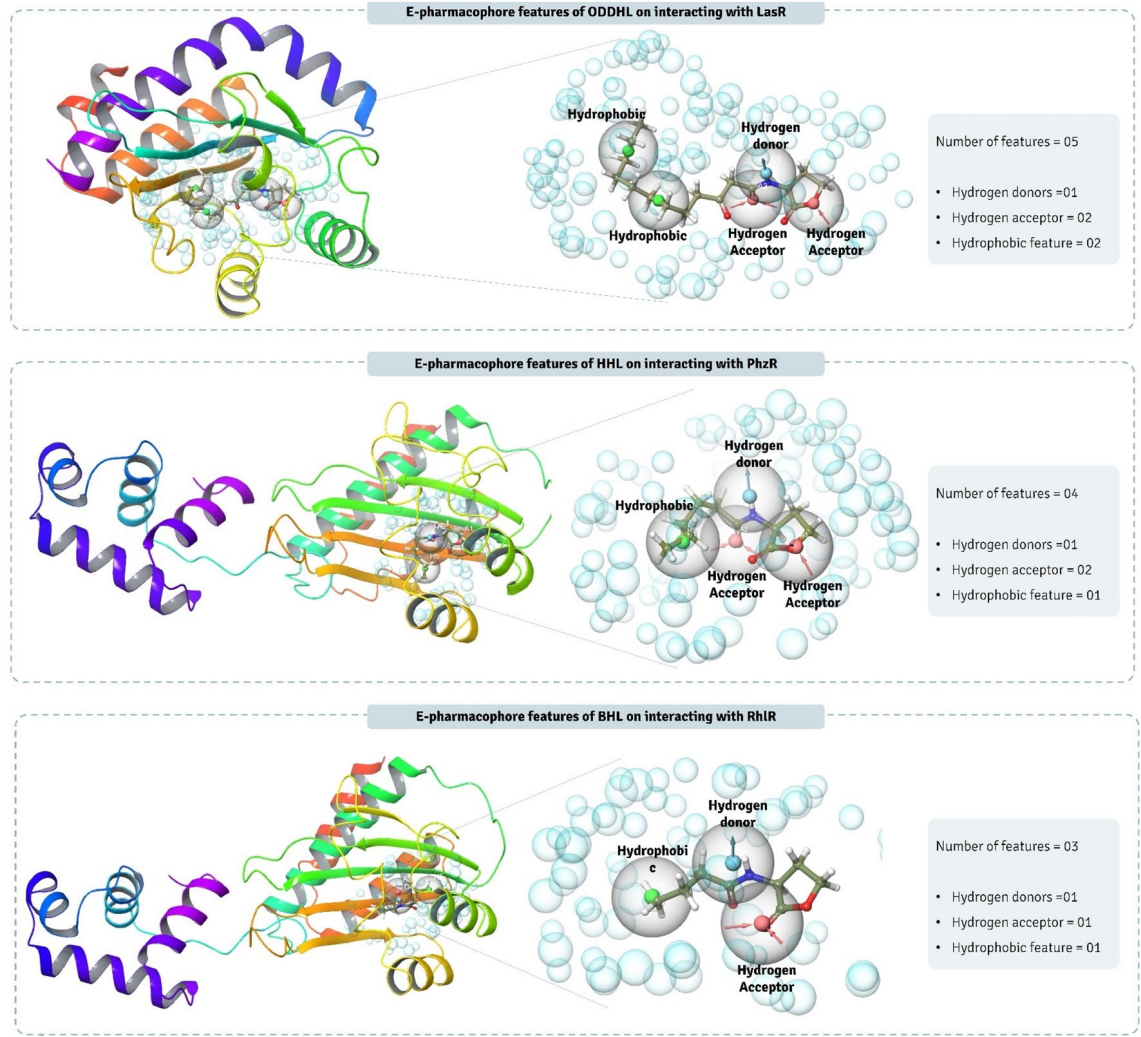
E-pharmacophore hypothesis of all the three complexes, LasR-ODDHL, PhzR-HHL and RhlR-BHL.

From 9500 phytochemicals of IMPPAT database, the aim is to identify molecules that can interact with all three proteins LasR, PhzR and RhlR to inhibit their function. For this, all three proteins were docked using HVTS protocol where for each protein top 100 hits were identified. It is not definite that all the 100 molecules for all the proteins were identical, where some were, and others weren’t. The next level of screening was based on identifying those ligands that resemble structurally and chemically similar to the native ligands of LasR, PhzR and RhlR and for this E-pharmacophore feature mapping based screening of compounds was performed. The E-pharmacophore feature maps of ODDHL interacting with LasR, HHL interacting with PhzR and BHL interacting with RhlR is represented in Fig. 3. ODDHL while interacting with LasR showed 05 features in total, where number of features for hydrogen donors, hydrogen acceptor, and hydrophobic property was 01, 02 and 02 respectively. For HHL interacting with PhzR, total pharmacophore features were found to be 04, where number of features for hydrogen donors, hydrogen acceptor, and hydrophobic property was 01, 02 and 01 respectively. Similarly, for BHL interacting with RhlR, total pharmacophore features were found to be 03, where number of features for hydrogen donors, hydrogen acceptor, and hydrophobic property was 01, 01 and 01 respectively (Fig. 3). Based on this E-pharmacophore featured for all the three ligand-receptor complexes, second level of screening was performed which identified 69 total compounds having identical features (Table 2). These 69 compounds had three native ligands (ODDHL, HHL and BHL) as reference while 66 hits were from IMPPAT database. The next step is to identify top 10 molecules from these 66 hits for each protein, and to achieve this all these compounds were first docked with each protein LasR, PhzR and RhlR with SP protocol, and top 50% of compounds (33 hits) were retained for next stage of docking with XP protocol. These 33 compounds were then docked again with each of these protein LasR, PhzR and RhlR, after which top 10 compounds were considered for further assessment. These top 10 compounds for each protein under study and their docking scores are represented in Table 3. MM-GBSA calculations was performed with these 10 compounds for each protein to predict the degree of spontaneity for their reactivity towards respective protein. The Gibbs free energy, ΔG, change determined by MM-GBSA for top 10 compounds for each protein is represented in Table 3. For LasR, top 10 molecules ranked best to worst, based on docking scores are Nb-p-Coumaroyltryptamine, Curcumin, 6-Gingerol, (-)-Epicatechin, Galangin, 7-Hydroxyflavanone, Capsaicin, Resveratrol, Shogaol, and N-Benzoyl-L-phenylalaninol. For PhzR, top 10 molecules ranked best to worst, based on docking scores are Curcumin, Irisolidone, 6-Gingerol, Baicalein, Fisetin, Kaempferol, Lupiwighteone, 7-Hydroxyflavanone, Capsaicin, and Liquiritigenin. Lastly, for RhlR, top 10 molecules ranked best to worst, based on docking scores are Capsaicin, 6-Gingerol, Curcumin, Resveratrol, Caffeic acid, Curcumin, Ferulic acid, Ajoene, (-)-Epicatechin gallate, and Methylenedioxybenzoyl ethyl PABA. From all these compounds, Capsaicin, 6-Gingerol, and Curcumin were found to be common for all these three proteins, though out of these top three compounds, Capsaicin had the poorest MM-GBSA profile and hence was eliminated. Structure comparison of gingerol with different AHLs suggests that gingerol is ~ 30% larger in size along with ~ 40% greater surface area when compared to the largest AHL (ODDHL). Moreover, AHL and gingerol share certain similar structural features. Gingerol possess a hydroxyl group at the 5-position, a carbonyl group at the 3-position, and a 4′-hydroxy-3′-methoxy phenyl group at 1-position of its decane backbone structure along with possessing a long alkyl chain showing analogy to AHLs. However, the terminal aromatic nature of 6-Gingerol distinguishes it from AHLs. In this way 6-Gingerol and Curcumin was identified were top two hits as QSA with which MD simulations and in vitro assessments were performed.

**Table 2:** Screened compounds based on E-pharmacophore feature mapping.

Screened compounds	PubChem CID	Features
OdDHL	3,246,941	A(1) A(4) D(5) H(7) H(6)
HHL	10,058,590	A(1) A(3) D(4) H(-) H(-)
BHL	10,130,163	A(1) A(3) D(4) H(-) H(-)
Moupinamide	5,280,537	A(-) A(2) D(7) H(8) H(-)
Methylenedioxybenzoyl ethyl PABA	785,868	A(1) A(4) D(6) H(7) H(-)
Kinetin riboside	20,345	A(8) A(3) D(12) H(-) H(-)
5-Hydroxy-6,7,4'-trimethoxyisoflavone	10,830,108	A(3) A(-) D(-) H(9) H(8)
Capsaicin	1,548,943	A(1) A(2) D(-) H(7) H(9)
Saussureamine C	9,998,735	A(2) A(5) D(9) H(-) H(10)
6-Gingerol	442,793	A(2) A(3) D(-) H(-) H(8)
Irisolidone	5,281,781	A(6) A(5) D(-) H(10) H(-)
Chrysoeriol	5,280,666	A(2) A(3) D(-) H(-) H(10)
4'-O-Methylkievitone	44,257,389	A(2) A(3) D(-) H(10) H(13)
Jaceosidin	5,379,096	A(3) A(4) D(-) H(-) H(12)
N-Benzoyl-L-phenylalaninol	100,005	A(1) A(2) D(4) H(-) H(-)
Batatifolin	5,320,181	A(2) A(3) D(-) H(-) H(12)
Broussonin C	442,289	A(3) A(2) D(-) H(7) H(10)
Ursolic acid	64,945	A(-) A(2) D(-) H(11) H(13)
Gancaonin P 3'methyl ether	5,317,483	A(1) A(6) D(-) H(12) H(15)
Dihydrozeatin riboside	10,522,005	A(6) A(2) D(9) H(15) H(14)
Chavicine	1,548,912	A(-) A(2) D(-) H(4) H(5)
Lupinalbin C	14,309,762	A(7) A(5) D(-) H(-) H(11)
Ovalitenone	627,910	A(2) A(5) D(-) H(-) H(8)
Licoisoflavone B	5,481,234	A(6) A(5) D(-) H(10) H(12)
Chlorogenic Acid.1	1,794,427	A(2) A(7) D(10) H(-) H(-)
Silibinin.1	31,553	A(9) A(7) D(-) H(16) H(-)
Gancaonin I	480,777	A(5) A(4) D(-) H(12) H(10)
BavachalconeCID_6450879.1	6,450,879	A(-) A(1) D(6) H(8) H(10)
Bavachin	14,236,566	A(4) A(1) D(-) H(8) H(9)
gallic acid_CID_370.1	370	A(1) A(3) D(8) H(-) H(-)
(-)-Epicatechin	72,276	A(3) A(1) D(7) H(-) H(-)
( +)-Isofebrifugine	11,208,839	A(4) A(2) D(6) H(-) H(-)
Leucopelargonidin	3,286,789	A(4) A(1) D(7) H(-) H(-)
Glycyrrhisoflavone	5,317,764	A(6) A(2) D(-) H(12) H(13)
Zingerone	31,211	A(2) A(1) D(4) H(-) H(-)
Moracin C	155,248	A(4) A(1) D(-) H(8) H(10)
Lupiwighteone	5,317,480	A(-) A(4) D(-) H(9) H(11)
Malvidin_min.1	159,287	A(5) A(6) D(-) H(12) H(-)
Ferulic acid_CID_445858.1	445,858	A(-) A(3) D(6) H(7) H(-)
6-shogaol.1	5,281,794	A(1) A(-) D(4) H(7) H(6)
( +)-Alangimaridine	10,853,265	A(-) A(2) D(5) H(9) H(7)
Resveratrol.1	445,154	A(-) A(2) D(6) H(-) H(7)
Licoflavonol	5,481,964	A(6) A(-) D(9) H(11) H(13)
Ajoene	5,386,591	A(-) A(-) D(-) H(2) H(3)
Arachidin-3	11,380,920	A(1) A(2) D(-) H(-) H(-)
Ellagic acid	5,281,855	A(7) A(3) D(-) H(-) H(-)
Curcumin	969,516	A(2) A(-) D(8) H(-) H(-)
7,4'-Dihydroxyflavan	158,280	A(-) A(-) D(5) H(-) H(6)
Dihydrobaicalein	9,816,931	A(5) A(1) D(-) H(-) H(-)
Equol	91,469	A(-) A(3) D(-) H(-) H(6)
Nb-p-Coumaroyltryptamine	5,458,878	A(2) A(-) D(-) H(-) H(6)
Barbaloin	12,305,761	A(-) A(-) D(13) H(-) H(17)
1,3,8-Trihydroxy-5-methoxy-xanthen-9-one	5,322,042	A(-) A(-) D(9) H(10) H(-)
taxifolin_min.1	439,533	A(3) A(-) D(8) H(-) H(-)
caffeine_min.1	2519	A(1) A(2) D(-) H(-) H(-)
Anhydroglycinol	442,667	A(-) A(3) D(-) H(-) H(7)
Cianidanol	9064	A(4) A(2) D(-) H(-) H(-)
caffeic acid	689,043	A(-) A(4) D(5) H(-) H(-)
Baicalein	5,281,605	A(1) A(2) D(-) H(-) H(-)
Kaempferol	5,280,863	A(1) A(2) D(-) H(-) H(-)
Galangin	5,281,616	A(1) A(2) D(-) H(-) H(-)
Apigenin	5,280,443	A(1) A(2) D(-) H(-) H(-)
Chrysin	5,281,607	A(1) A(2) D(-) H(-) H(-)
Fisetin	5,281,614	A(-) A(4) D(9) H(-) H(-)
Epicatechin gallate	107,905	A(9) A(-) D(15) H(-) H(-)
Liquiritigenin	114,829	A(4) A(1) D(-) H(-) H(-)
7-Hydroxyflavanone	1890	A(3) A(2) D(-) H(-) H(-)
Dihydronorwogonin	42,608,113	A(-) A(5) D(6) H(-) H(-)

*A* hydrogen acceptor, *D* hydrogen donor, and *H* hydrophobicity.

**Table 3:**
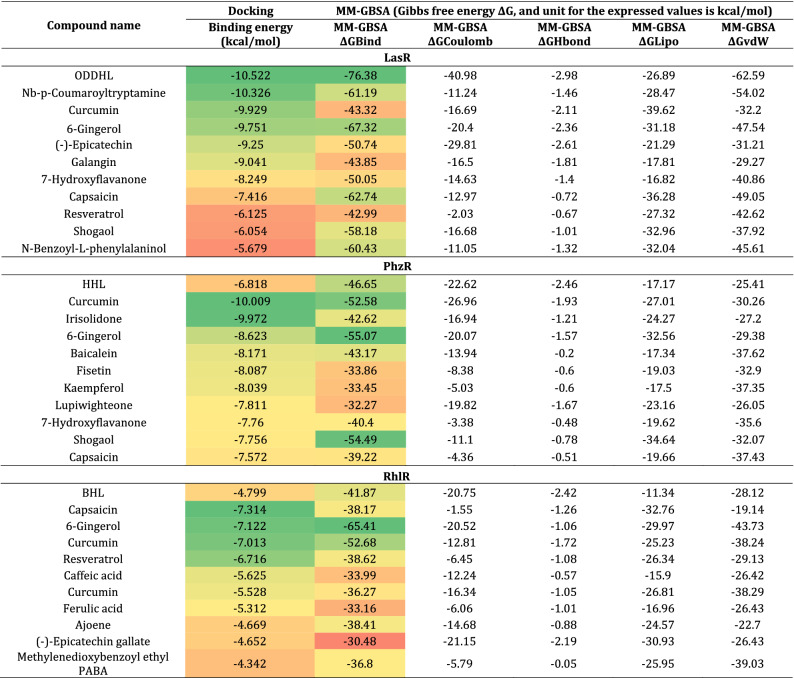
Molecular docking and MM-GBSA energy profiles of native ligands and screened top hits during their interaction with LasR, PhzR and RhlR.

*ΔGBind =* binding energy, *ΔGCoulomb =* Coulomb energy, *ΔGHbond =* hudrogen-bonding correction, *ΔGLipo =* lipophilic energy, *ΔGvdW =* Van der Waals energy.

### Assessing the impact of screened QSAs on growth of *P. aeruginosa*

**Figure 4:**
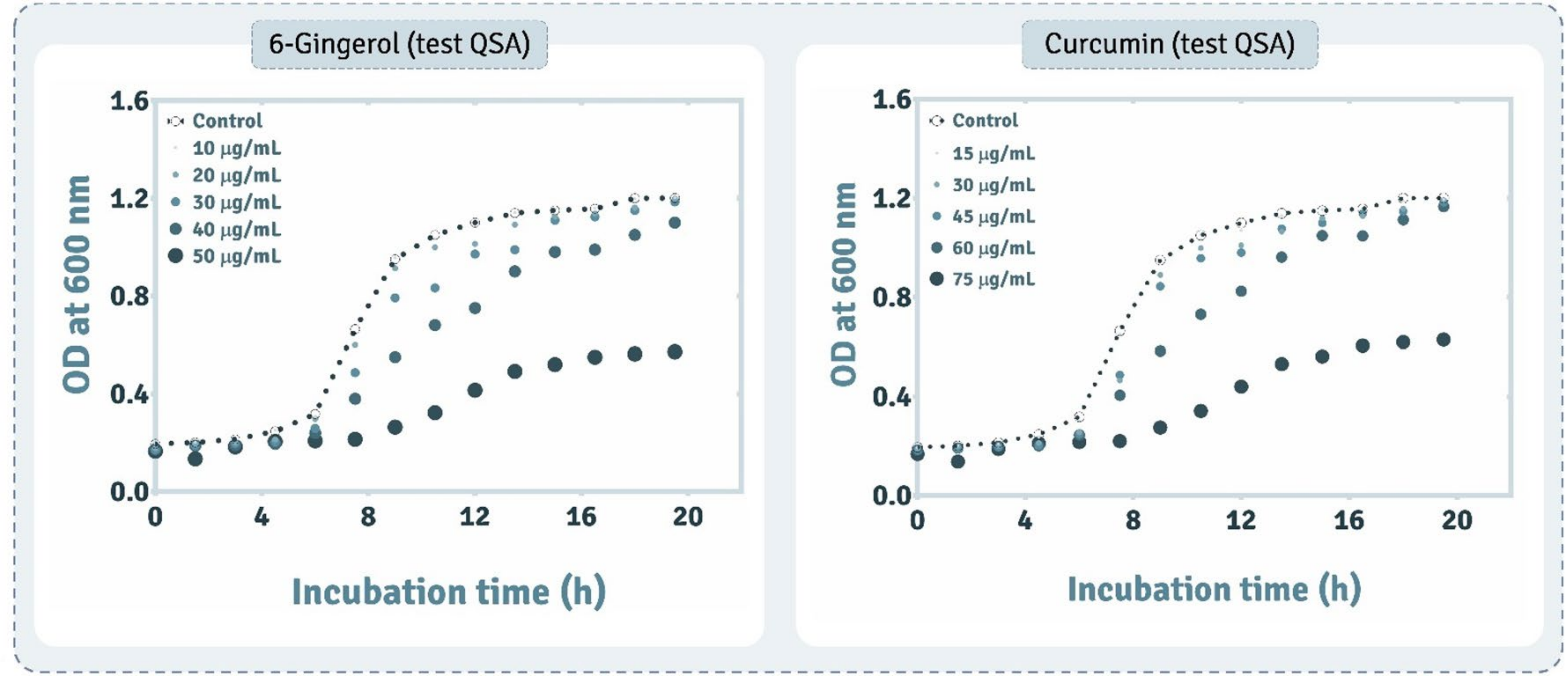
Assessing the influence on growth of *P. aeruginosa* in presence of varying concentrations of (**a**) 6-Gingerol and (**b**) Curcumin.

The influence of screened and docked compounds, 6-Gingerol and Curcumin, on the growth of *P. aeruginosa* AM26 was performed to determine a threshold concentration of these compounds beyond which the increase in their concentration would lead to a significant reduction in the biomass. Hence, the growth assessment was performed by allowing the organism to grow in a medium supplemented with 5–30 µg/mL of screened QSAs. It can be observed from Fig. 4 that when the organism is not treated with any QSA, at sixth hour the culture enters the logarithmic growth phase whereon there is a steep increase in the biomass which stabilizes at around eleventh hour. The same trend of culture growth in presence of 6-Gingerol was retained up to 20 µg/mL beyond the slope of logarithmic phase was deviating to the lower values. It was observed that at eleventh hour 30 µg/mL of 6-Gingerol showed ~ 10% decrease in the overall cell turbidity while at 40 µg/mL the decrease was ~ 35% and at 50 µg/mL concentration ~ 70% decrease in cell turbidity was observed. For Curcumin, at 45 µg/mL, 60 µg/mL, and 75 µg/mL concentrations the reduction in the cell turbidity observed was ~ 12%, 25%, and 60% respectively. From the growth study it can deduced that 6-Gingerol and Curcumin had inhibitory or growth suppressive influence on the cells of *P. aeruginosa* AM26. 30 µg/mL concentration of 6-Gingerol was considered the threshold concentration which was maximally tolerated by the organism without its growth being affected. So, for 6-Gingerol 30 µg/mL concentration was considered a critical concentration where inhibition in QS pathways be evaluated without their growth being significantly altered. Further, the same concentration (30 µg/mL) was taken for 6-Gingerol for assessing changes in the antibiotic susceptibility assay. Similarly, for Curcumin the threshold concentration which did not affect the growth characteristics was found to be 45 µg/mL. Therefore, this concentration for Curcumin was used for antibiotic susceptibility assay.

### Assessment of LasR interactions with screened QSAs

Here we determined (i) how effectively can QSAs, 6-Gingerol and Curcumin interact with LasR, and for this we performed extensive computational assessment corelating the findings of docking with MD simulations, and (ii) how these QSAs at sub-MIC concentrations modulate the phenotypic trait of EPS and biofilm production which are driven directly by LasR dependent QS pathway.

**Figure 5:**
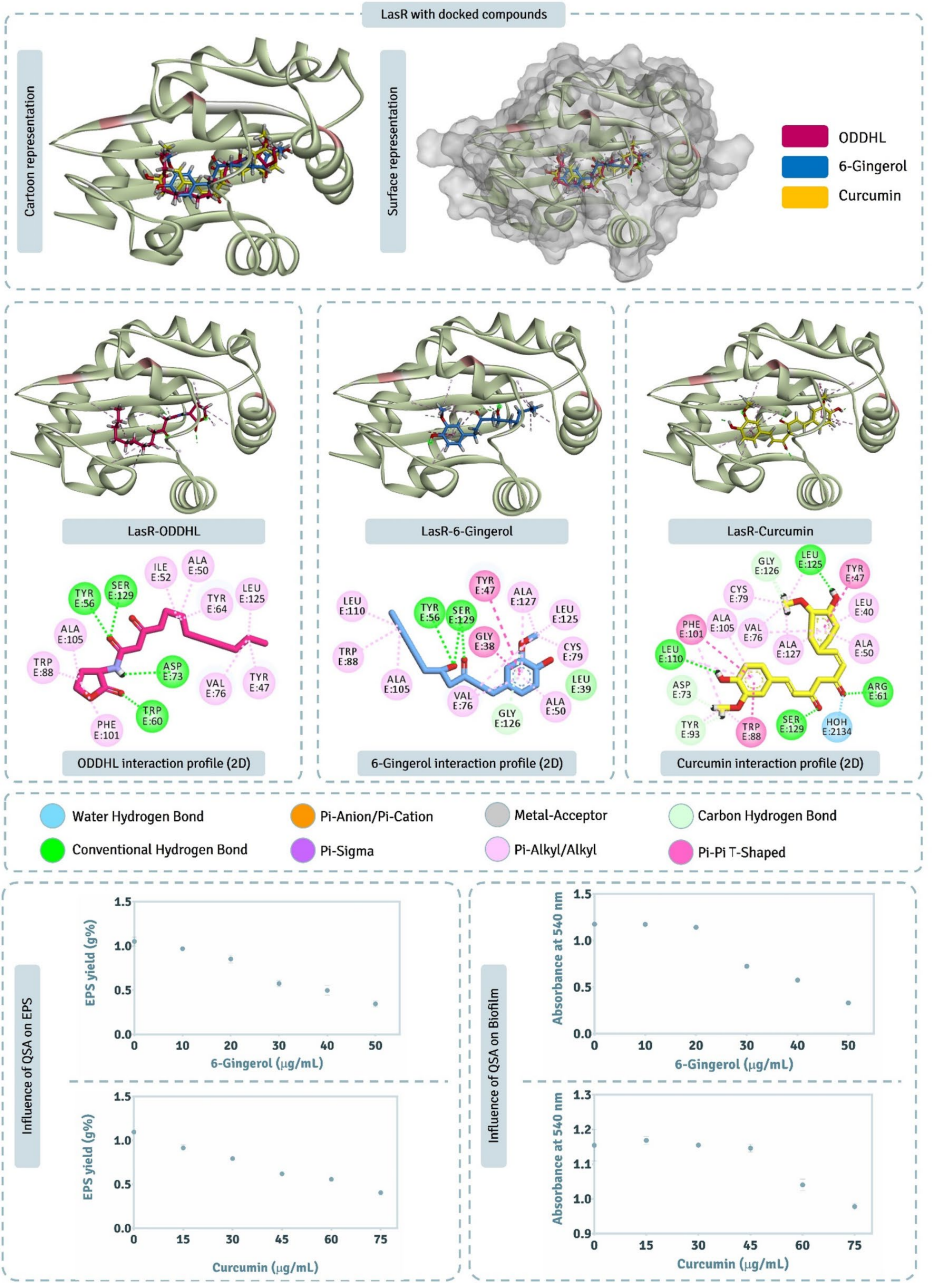
Interaction profile of LasR with 6-Gingerol and Curcumin as predicted by XP docking and assessment of its QSA potential on EPS and Biofilm production.

The detailed interaction of ODDHL (native AHL of LasR), and two QSAs (6-Gingerol and Curcumin) with LasR obtained by XP docking protocol is represented in Fig. 5. The native ligand, ODDHL, interacts with Tyr56, Trp60, Asp73 and Ser129 by making Hydrogen bonds, while interacts with Tyr47, Ala50, Ile52, Tyr64, Val76, Trp88, Phe101, Ala105 and Leu125 by making Pi-Alkyl interactions, in total this ligand interacts with 13 amino acids out of 26 amino acids that constitute active site cavity as per CASTp 3.0 assessment (Table 1). Docking score for this ligand was found to be − 10.522 kcal/mol and MM-GBSA assessment showed the ΔGBind to be − 76.38 kcal/mol (Table 3). Out of QSAs under study, Curcumin showed the docking of − 9.929 kcal/mol and for 6-Gingerol the docking energy obtained was − 9.751 kcal/mol, here both the compounds showed identical docking energies, but MM-GBSA assessment showed 6-Gingerol to have greater ΔGBind energies (− 43.32 kcal/mol) than that of Curcumin (− 67.32 kcal/mol) (Table 3) suggesting 6-Gingerol to interact more spontaneously than Curcumin even when their docking energies being identical. 6-Gingerol interacted with 14 amino acids in the active site of LasR, of which it interacted Tyr56 and Ser129 by forming hydrogen bonds, Leu39 and Gly126 by forming Carbon-Hydrogen bond, Gly38 and Tyr47 by forming Pi-Pi T-shaped hydrophobic interactions and Ala50, val76, Cys79, Trp88, Ala015, Leu110, Leu125 and Ala127 by forming Pi-Alkyl interactions. Curcumin interacted with 17 amino acids in the active site of LasR, the details of interactions so formed by each ligand explain here is shown in Fig. 5.

**Figure 6:**
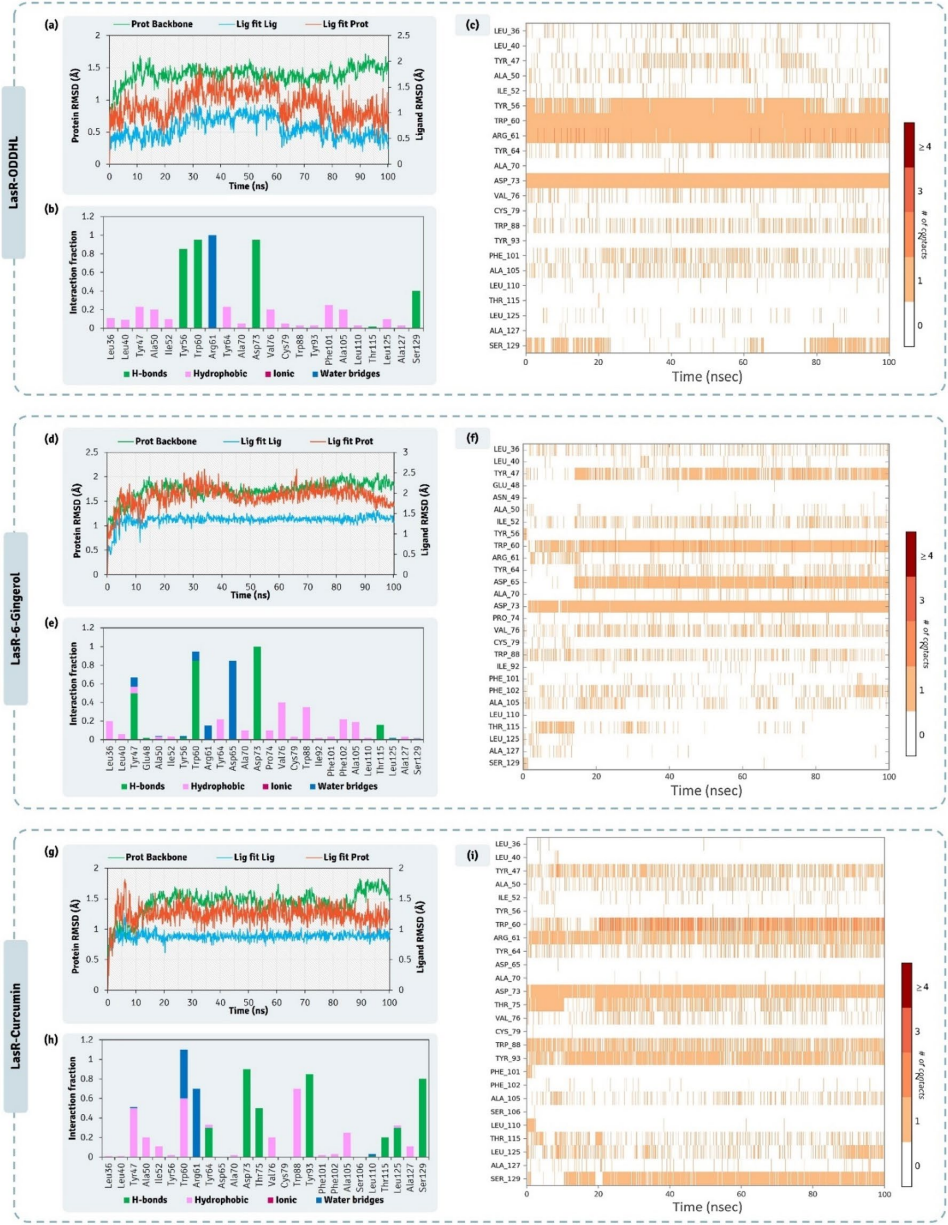
100 ns MD simulation trajectory representing the RMSD assessment, ligand-receptor interaction profile and ligand-receptor interaction timeline for LasR-ODDHL, LasR-6-Gingerol, and LasR-Curcumin complexes.

A 100 ns MD simulation were performed for the complexes, (i) LasR-ODDHL (iii) LasR-6-Gingerol and (iii) LasR-Curcumin, to consolidate the findings of docking. RMSD, and protein–ligand contact profiles for their types and their interaction time profiles all the MD trajectories were calculated. 6-Gingerol and Curcumin were identified to be the best hits as QSAs, so LasR-6-Gingerol and LasR-Curcumin MD runs were considered as the test while the MD run of LasR-ODDHL was considered as reference control. The RMSD of individual molecules in the protein ligand complex and their movements with respect to each other is depicted in Fig. 6. The RMSD values in the left Y-axis denotes the vales of protein and the right Y-axis represents the vales of ligand. For the protein assessment only the value on the left Y-axis is to be seen where the values up to 3.0 Å is considered ideal, while the values above 3 Å represents the conformation change in the 3D architecture of the protein. Here, it was observed that the 3D architecture of LasR being stable in presence of all the three ligands ODDHL (Fig. 6a), 6-Gingerol (Fig. 6d) and Curcumin (Fig. 6g), none of these complexes appear to be exceeding the protein RMSD beyond 2.5 Å. Additionally, Ligand RMSD represented in the right Y-axis, describes the movement of ligand with respect to the protein (Lig fit Prot). Also, the individualistic RMSD of ligand is described as ‘Lig fit Lig’ in the RMSD plots. The ‘Lig fit Prot’ values close to protein RMSD suggests equal movement of protein backbone and ligand. Ligands being much smaller in size than the protein often demonstrate the ‘Lig fit Prot’ values higher than protein RMSD. However, two to three folds higher values of ‘Lig fit Prot’ than that of protein RMSD suggests the ligand is changing poses and orientations in the protein cavity to attain stable spatial arrangement. In this study, the ‘Lig fit Prot’ values for all the three ligands doesn’t exceed by two folds with respect to the corresponding protein RMSD values. Moreover, the ‘Lig fir Prot’ values of 6-Gingerol (Fig. 6d) and Curcumin (Fig. 6g) was identical to that of reference, ODDHL (Fig. 6a) suggesting that poses of both the QSA predicted by docking was appropriate as their ‘Lig fit Prot’ values is identical to that of the co-crystalized ligand ODDHL (Fig. 6a). The interaction types exhibited by ligand, ODDHL with LasR is represented in Fig. 6b, 6-Gingerol with LasR is represented in Fig. 6e, Curcumin with LasR is represented in Fig. 6h. In these charts the interaction fraction represents the normalized cumulative interaction profile: for example, an assessment of 0.8 suggests that 80% of the time during the simulation, the corresponding interaction remains durable. Value over 1.0 are possible as some amino acids may have more than one type of contact of the equivalent subtype with the ligand. While ligand interaction timeline of ODDHL, 6-Gingerol, and Curcumin with LasR during MD simulation is represented in Fig. 6c,f,i, from the entire study of MD simulation it is evident the interaction of all the ligands with LasR is stable and poses predicted by docking are apt for ideal interaction.

Figure 5 represents the impact of QSAs on EPS and biofilm where, it is observed that 6-Gingerol and Curcumin both have an inverse relationship with EPS and biofilm production. At the critical threshold concentration for 6-Gingerol (30 μg/mL) there was ~ 50% reduction in EPS yield and ~ 40% reduction in the biofilm formation. As mentioned above, this concentration of 6-Gingerol does not influence the growth of *P. aeruginosa* AM26 but here, at this concentration there was considerable reduction in both EPS and biofilm production suggesting suppression in LasR mediated QS. For Curcumin, the critical threshold concentration, as mentioned above, was 45 μg/mL and at this concentration ~ 35% reduction in EPS yield and < 10% reduction in the biofilm production suggesting suppression in LasR mediated QS pathways but less in magnitude than seen with 6-Gingerol.

### Assessment of PhzR interactions with screened QSAs.

Here we determined (i) how effectively can QSAs, 6-Gingerol and Curcumin interact with PhzR, was performed using computational assessments, and (ii) how these QSAs at sub-MIC concentrations modulate the phenotypic trait of Pyocyanin production which is driven directly by PhzR dependent QS pathway.

**Figure 7:**
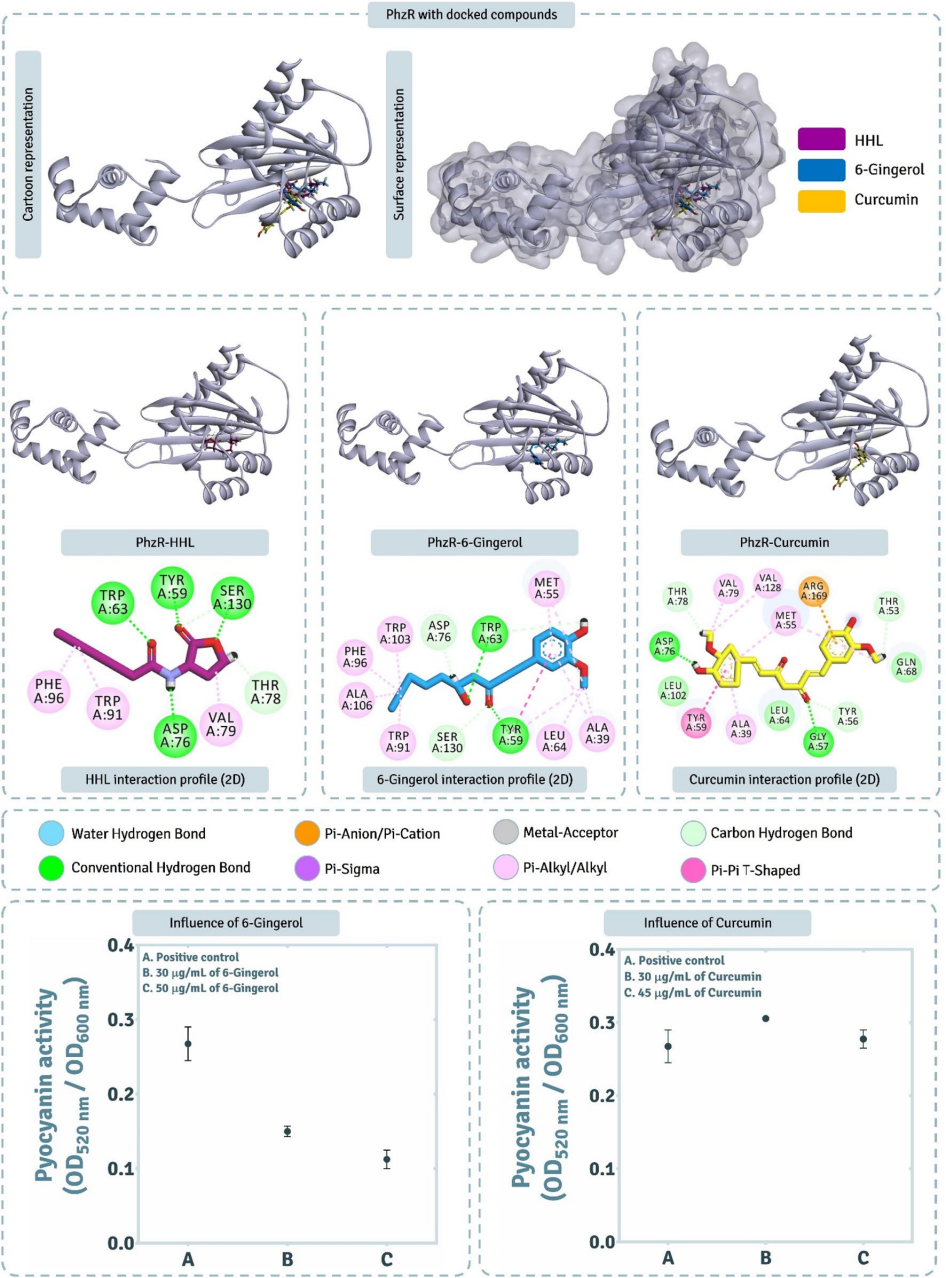
Interaction profile of PhzR with 6-Gingerol and Curcumin as predicted by XP docking and assessment of its QSA potential on pyocyanin activity.

The detailed interaction of HHL (native AHL of LasR), and two QSAs (6-Gingerol and Curcumin) with PhzR obtained by XP docking protocol is represented in Fig. 7. Docking scores of HHL, 6-Gingerol and Curcumin with PhzR was found to be − 6.818 kcal/mol, − 8.623 kcal/mol and − 10.009 kcal/mol respectively (Table 3). Docking assessment showed both Curcumin and 6-Gingerol both had much better interacting ability than native ligand HHL as per theoretical XP docking protocol. MM-GBSA assessment showed the ΔGBind energies for HHL, 6-Gingerol and Curcumin to be − 46.65 kcal/mol, − 55.07 kcal/mol and − 52.58 kcal/mol respectively (Table 3). ΔGBind energies predicted the spontaneity for 6-Gingerol and Curcumin to be identical, while being even better than native ligand, HHL. HHL showed to interact with Tyr59, Trp63, Asp76, Thr78, Val79, Trp91, Phe96 and Ser130 (Fig. 7). 6-Gingerol showed to interact with Ala 39, Met55, Tyr59, Trp63, Leu64, Asp76, Thr78, Val79, Trp91, Phe96, Trp103, Ala106 and Ser130 (Fig. 7). Curcumin showed to interact with 14 amino acids (Fig. 7) out of 26 amino acids of active site predicted by CASTp 3.0.

**Figure 8:**
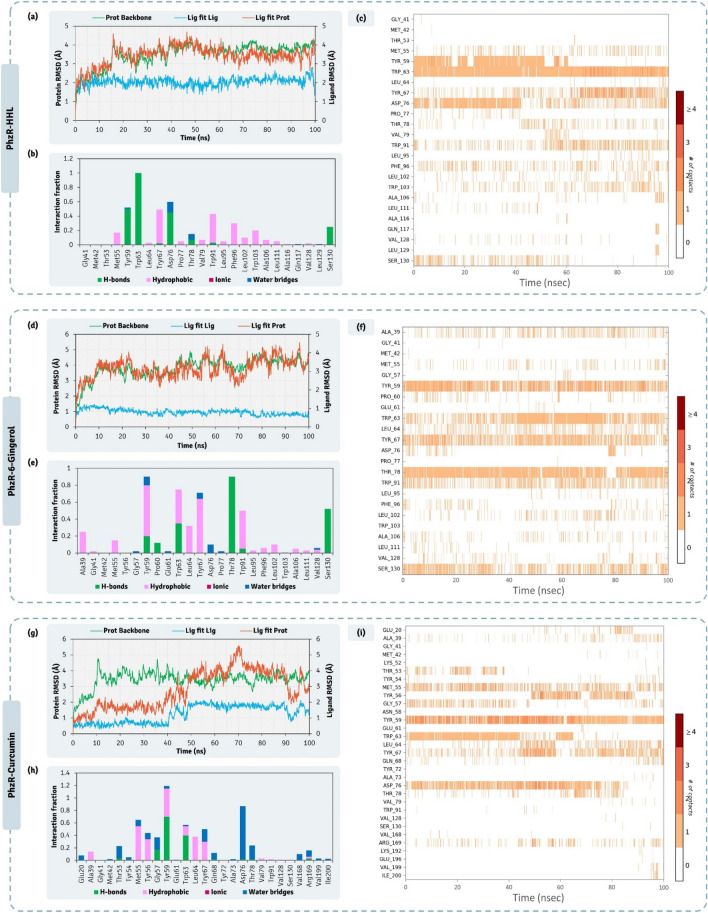
100 ns MD simulation trajectory representing the RMSD assessment, ligand-receptor interaction profile and ligand-receptor interaction timeline for PhzR-HHL, PhzR-6-Gingerol, and PhzR-Curcumin complexes.

From the 100 ns MD simulations of complexes PhzR-HHL, PhzR-6-Gingerol, and PhzR-Curcumin, RMSD assessments for protein interacting with ligands HHL (Fig. 8a), 6-Gingerol (Fig. 8d) and Curcumin (Fig. 8g), protein RMSD peaked up to 4.5 Å, which is higher than previously seen with LasR, this is due to the movement in the HTH region of the PhzR which is highly flexible and not found in LasR. This does not seem to be an issue as the RMSD value for protein while interacting with native ligand, which is considered as reference control peaks to 4.0 Å as well. ‘Lig fit Prot’ for HHL, 6-Gingerol and Curcumin peaks at 4.5 Å (Fig. 8a), 4.5 Å (Fig. 8d) and 5.5 Å (Fig. 8g) respectively. For Curcumin, the ‘Lig fit Prot’ values suddenly peak from 4.0 Å to 5.5 Å at ~ 70 ns and then again stabilizes to 4.0 Å at ~ 75 ns. This shows the ligand Curcumin changing orientation to acquire a more stable pose. The interaction types exhibited by ligand, HHL with PhzR is represented in Fig. 8b, 6-Gingerol with PhzR is represented in Fig. 8e, Curcumin with PhzR is represented in Fig. 8h. Here the pattern of interaction exhibited by ligands is identical to the ones predicted by XP docking assessment represented in Fig. 7. While ligand interaction timeline of HHL, 6-Gingerol, and Curcumin with PhzR during MD simulation is represented in Fig. 8c,f,i, from the entire study of MD simulation it is evident the interaction of all the ligands with PhzR is stable and poses predicted by docking are apt for ideal interaction.

The effect of QSAs on pyocyanin production (Fig. 7) suggests that at threshold tolerated concentration of 6-Gingerol, 30 μg/mL, there was ~ 30% reduction in pyocyanin activity while for Curcumin for its threshold tolerated concentration of 45 μg/mL concentration, no reduction in pyocyanin production was observed. This suggests that even when Curcumin showed promising interaction with PhzR under in silico assessment, its treatment on *P. aeruginosa* AM26 for assessing PhzR mediated QS pathway was unaltered.

### Assessment of RhlR interactions with screened QSAs.

Here we determined (i) how effectively can QSAs, 6-Gingerol and Curcumin interact with RhlR, was performed using computational assessments, and (ii) how these QSAs at sub-MIC concentrations modulate the phenotypic trait of Rhamnolipid production which is driven directly by RhlR dependent QS pathway.

**Figure 9:**
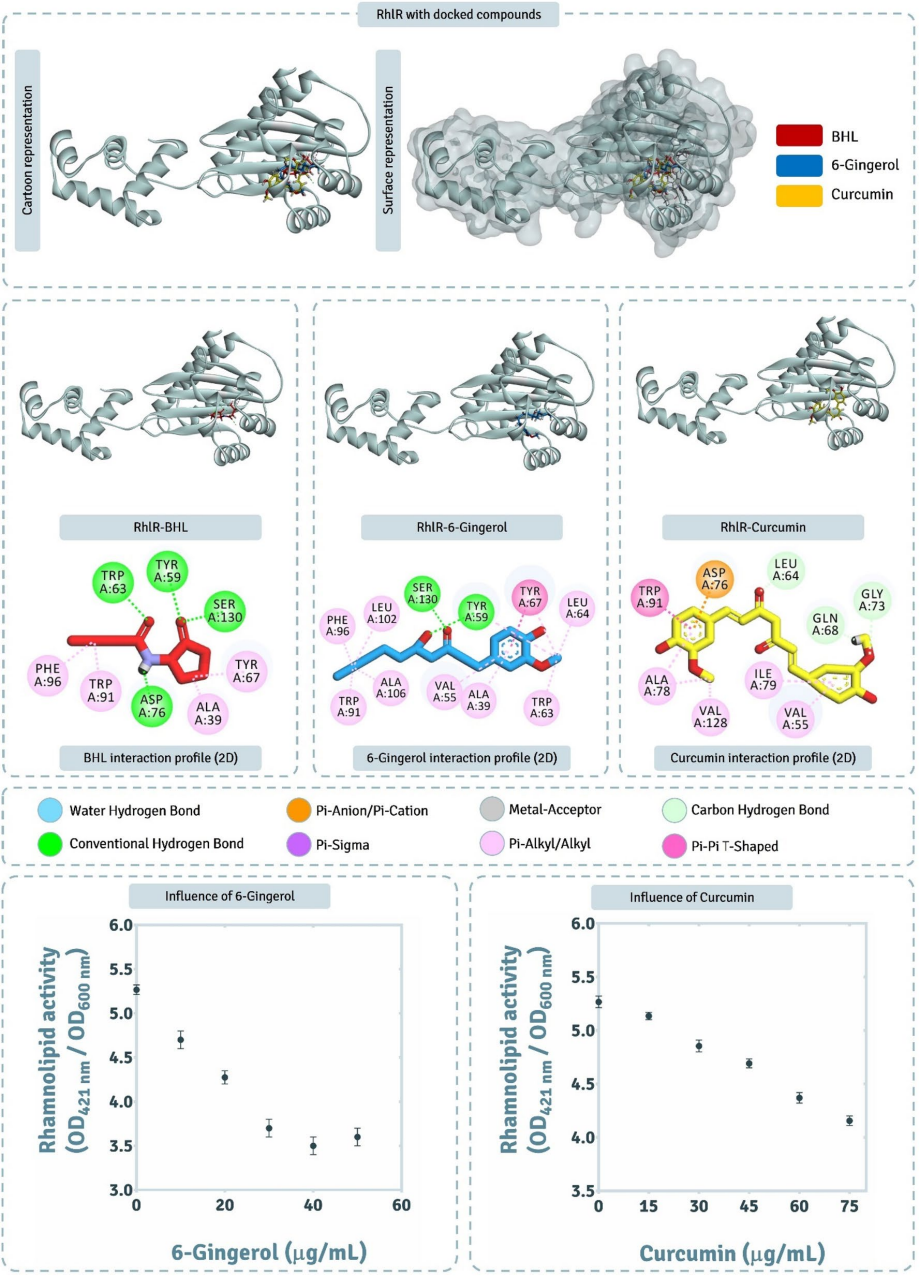
Interaction profile of RhlR with 6-Gingerol and Curcumin as predicted by XP docking and assessment of its QSA potential on rhamnolipid activity.

The detailed interaction of BHL (native AHL of LasR), and two QSAs (6-Gingerol and Curcumin) with RhlR obtained by XP docking protocol is represented in Fig. 9. The active site of this protein is found to be the largest of all as per the protein topology assessment by CASTp 3.0 and consists of 31 amino acids. The native ligand, BHL interacts with Trp59, Trp63, Asp76 and Ser13 by making hydrogen bonds, while with Ala39, Tyr67, Trp91 and Phe96 by forming Pi-Alkyl interactions. 6-Gingerol interacted with Tyr59 and Ser130 by making hydrogen bonds; Tyr67 by making Pi-Pi T-shaped interaction, while with Ala39, Val55, Trp63, Leu64 Trp91, Phe96, Leu102 and Ala106 by making Pi-Alkyl interactions. Curcumin for this protein showed poor interaction profile when compared to its interaction profile with previous two proteins LasR and PhzR. Here Curcumin fails to make any hydrogen bond, but makes Pi-Anionic bond with Asp76, carbon-hydrogen bonds with Leu64, Gln68, Pi-Pi T-shaped interaction with Trp91, and Gly73, and Pi-Alkyl interactions with Val55, Ala78, Ile79 and Val128. Docking scores of BHL, 6-Gingerol and Curcumin with RhlR was found to be − 4.790 kcal/mol, − 7.122 kcal/mol and − 7.013 kcal/mol respectively (Table 3), suggesting both the screened QSAs to interact with even better affinity than showed by native ligand BHL. The spontaneity of interaction predicted as per MM-GBSA showed 6-Gingerol to be superior than Curcumin, where the ΔGBind for 6-Gingerol was found to be − 65.41 kcal/mol, while − 52.68 kcal/mol for Curcumin (Table 3). Thus, for this protein 6-Gingerol showed the edge over Curcumin based on their interaction forming capabilities as QSAs.

**Figure 10:**
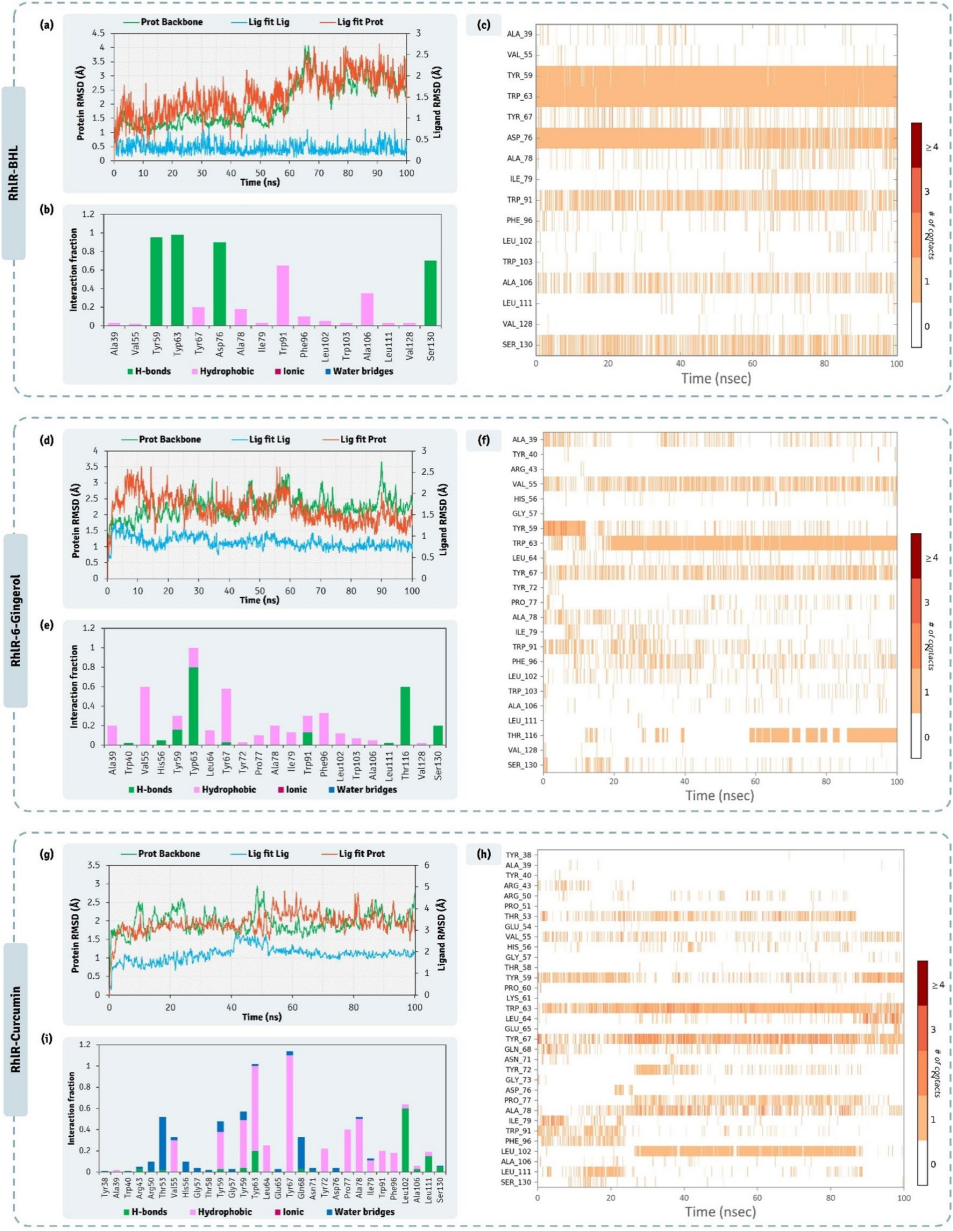
100 ns MD simulation trajectory representing the RMSD assessment, ligand-receptor interaction profile and ligand-receptor interaction timeline for RhlR-HHL, RhlR-6-Gingerol, and RhlR-Curcumin complexes.

From the 100 ns MD simulations of complexes RhlR-BHL, RhlR-6-Gingerol, and RhlR-Curcumin, RMSD assessments for protein interacting with ligands BHL (Fig. 10a), 6-Gingerol (Fig. 10d) and Curcumin (Fig. 10g). The protein backbone RMSD for the protein interacting with all the three ligands never exceeded 4.0 Å, suggesting the protein remaining stable while interacting with all the three ligands. ‘Lig fit Prot’ for BHL, 6-Gingerol and Curcumin peaks at 2.7 Å (Fig. 10a), 2.5 Å (Fig. 10d) and 5.0 Å (Fig. 10g) respectively. For Curcumin the ‘Lig fit Prot’ values being ~ 50% greater to that obtained for BHL and 6-Gingerol suggests its poor stability. The interaction types exhibited by ligand, BHL with RhlR is represented in Fig. 10b, 6-Gingerol with RhlR is represented in Fig. 10e, Curcumin with RhlR is represented in Fig. 10h. Here the pattern of interaction exhibited by ligands is at identical to the ones predicted by XP docking assessment represented in Fig. 9. While ligand interaction timeline of BHL, 6-Gingerol, and Curcumin with RhlR during MD simulation is represented in Fig. 10c,f,i, from the entire study of MD simulation it is evident the interaction of all the ligands with RhlR is stable, except Curcumin and poses predicted by docking are apt for ideal interaction.

In rhamnolipid production (Fig. 9), the effect of 6-Gingerol and Curcumin at their respective threshold concentrations of 30 μg/mL and 45 μg/mL, there was a reduction by ~ 50% and ~ 20%. From the results obtained so far for 6-Gingerol and Curcumin, despite being predicted to effectively interact with all the AHLr of *P. aeruginosa*, 6-Gingerol showed better suppression than Curcumin when assessed biochemically for in vitro assays. Further, 6-Gingerol was found to be more effective at lower concentrations as compared to Curcumin for suppression of QS pathways.

### Antibiotic susceptibility modulation in *P. aeruginosa* by QSAs

**Figure 11:**
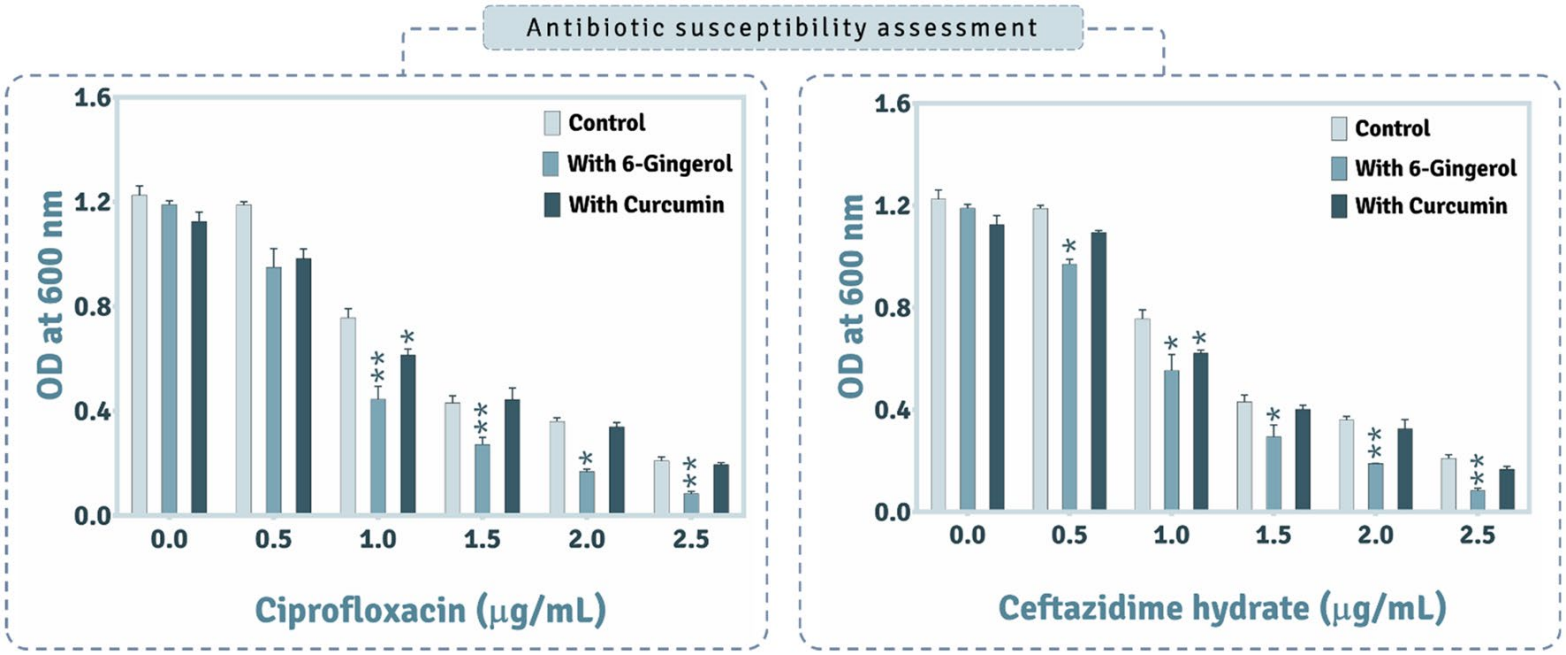
Assessing the impact of 6-Gingeol and Curcumin on modulation in antibiotic susceptibility of P. aeruginosa AM26 against ciprofloxacin and ceftazidime hydrate (n = 4, error bars, standard error of mean, Student T-test P value (control vs. test) represents the following ‘**’ for P < 0.00001, and ‘*’ for P < 0.0005 versus the control. *).

Figure 11 depicts the influence posed by QSAs on antibiotic tolerance by *P. aeruginosa* whereby which there was modification in the antibiotic susceptibility. The entire study of antibiotic susceptibility was performed at 30 μg/mL of 6-Gingerol and 45 μg/mL of Curcumin as these concentrations it was found that the growth of the culture was not impeded while there was effective reduction in QS. Here we tested two antibiotics Ciprofloxacin and Ceftazidime hydrate in combinations with varying concnetration of QSIs on *P. aeruginosa.* For Ciprofloxacin, it was observed that there was > 80% reduction in the cell density at 2.5 μg/mL concentration while at 0.5 μg/mL of Ciprofloxacin no inhibition in the growth was observed. 50% reduction in growth was observed at around 1.25 μg/mL which served as the minimum inhibitory concentration (MIC) for this antibiotic. In the set of experiments where, the culture was allowed to grow in presence of both, the varying concentrations of the antibiotic and, constant concentration (30 μg/mL) of 6-Gingerol, it was observed that for the concentration range from 0.5 μg/mL to 2.5 μg/mL, the effectiveness of antibiotic was improved. For instance, when the culture was treated with only 1 μg/mL of Ciprofloxacin the culture turbidity was ~ 0.8 OD and in the another set where the culture was grown in presence of 1 μg/mL Ciprofloxacin and 30 μg/mL of 6-Gingerol, the culture turbidity was ~ 0.45 OD which showed that the antibiotic susceptibility had improved by almost 40% in this case. Similarly, for Curcumin at this 1.0 μg/mL concentration of Ciprofloxacin, there was ~ 15% improvement in antibiotic susceptibility however, at other concentrations of Ciprofloxacin this difference was not as substantial as seen for 6-Gingerol. For another antibiotic, Ceftazidime hydrate, the pattern of inhibition was identical as that for Ciprofloxacin and the results here too showed 6-Gingerol to be more effective in improving the antibiotic susceptibility than Curcumin.

Thus, from all the in silico as well as in vitro assays it can deduced that: (i) 6-Gingerol and Curcumin both showed promising binding abilities with AHLr of *P. aeruginosa* AM26, (ii) 6-Gingerol was more effective in inhibiting the production of EPS, biofilm, pyocyanin, and rhamnolipid which were the phenotypes controlled by QS pathways, suggesting the inhibition of QS, (iii) Supplementation of 6-Gingerol was found to be more effective in improving potency of the antibiotics, Ciprofloxacin and Ceftazidime hydrate, against the microbe than Curcumin.

## Discussion

*Pseudomonas aeruginosa* is the leading cause of nosocomial infections in immunocompromised patients over and above being the causative agent for pneumonia, urinary tract, gastrointestinal, and skin infections. The prevalence of this microbe is attributed to its ability to produce biofilm in the hospital equipment like catheters, endoscopes, sonography probes etc. whereby the bacterium has easy access to the immunocompromised patients thereby making this opportunistic pathogen a contemporary nuisance organism. Moreover, this microbe has paved to thrive in such patients even under an antibiotic regime thus, making it to acquire resistance to multiple modern antibiotics. Therefore, the need of the hour is to treat this microbe with an alternative strategy which has unique mode of action not dealt so far. In the metaphorical impenetrable fort of *P. aeruginosa*, QS serves as a breakable window to shatter the microbial metabolic architecture thereby weakening it from within ultimately making it more vulnerable against routinely used control agents. Based on this rationale, we sought to identify promising naturally available bioactive compounds that can inhibit the QS machinery in *P. aeruginosa*. We made use of IMPPAT database which consists of bioactive compounds from Indian medicinal plants. In India, traditionally used spices also belong to these medicinal plants. One such unique example is the use of garlic in blocking QS in *P. aeruginosa* is well reported^29^. Further, garlic also shows antimicrobial properties, however, there are several phytochemicals with antimicrobial properties already identified with their mode of action still unknown. Joining all the dots, the question that sparked is that, Can the phytochemicals with antimicrobial properties can also serves QSA? To answer this question, we took the wholesome approach to screen an entire database consisting of bioactive compounds from Indian medicinal plants to specifically look at their affinity to interact AHLr which was made possible using computational studies and then validated the top hits using in vitro studies.

To perform such computational study, the prerequisite is to have the 3D models of crystallized AHLr, for this case of *P. aeruginosa* LasR, PhzR, and RhlR. LasR has been successfully crystallized and its structure is available in Protein DataBank (PDB) however, for other two proteins PhzR and RhlR structures are not available and therefore, PhzR and RhlR were modelled. In Gram-negative microbes, the QS receptors and signals bear high degree of identicality whereby all the AHLr are broadly classified into a single family of proteins known as “LuxR-family” which is vividly explained by. Under present study, RhlR and PhzR were modelled based on the available templates of LuxR family of protein and their quality were examined using QMEAN4, GMQE and Ramachandran plot analysis by MolProbity assessment. The results obtained showed the quality of the model built was at par with the parameters for homology modelling published so far.

Under current study, to screen large libraries of compounds based on the structure of the native signalling ligand molecule (AHLs) we made use of e-pharmacophore feature ligand mapping procedure to identify molecules having identical features such as H donor, H acceptor, aromatic, anionic, cationic, and hydrophobic. This also known as structure based primary screening of compounds which ultimately helps us to identify molecules with identical chemico-structural properties. Once we identify molecules with similar chemical behaviour to that of the native ligand, such compounds are then subjected for much robust extra precision (XP) docking to further filter compounds that can interact with target protein. To assess another set of criteria for these screened compounds is to identify how thermodynamically favourable the interactions between target protein and ligand is occurring, for which MM-GBSA assessment is performed which provides the ΔG’ (Gibbs free energy change) during the interaction between ligand and protein. By this we can pinpoint those phytochemicals that can spontaneously interact with the protein, in this case, LasR, PhzR, and RhlR. To verify whether the structural and pose integrity of the ligand on interacting with protein as predicted by docking persist in nature or not is predicted by performing MD simulations. This is the most robust assessment and validation for the interaction of ligand with protein and how accurate molecular docking has predicted the interaction. In brief, this pipeline of in silico assessment making using of e-pharmacophore feature mapping to screen structure-based ligand followed by docking, MM-GBSA and MD simulations is the widely accepted methodology in the domain of in silico studies for identifying potential hits for any given protein.

6-Gingerol and Curcumin were identified as top hits but along with these compounds, Epicatechin, Curcumin, Galangin, 7-Hydroxyflavanone, Capsaicin, Resveratrol, Shogaol, Zingerone, Kaempferol, Resveratrol, Caffeic acid, Ferulic acid, Ajoene, and Batatifolin also showed promising interactions with some but not all AHLr under study.

Epicatechin is a flavonoid commonly found in tea leaves and there are reports suggesting its antibacterial activity against *Helicobacter pylori*, *Staphylococcus aureus*, *Pseudomonas aeruginosa*, and *Candida albicans*. Moreover, recently it has been identified to serve as QSA in *P*. *aeruginosa*^28,30–34^. Galangin is flavanol found in several medicinal plants and has a rich literature on its antibacterial activity against *S. aureus*^35^. There are evidences for this compound to interfere natural microbial QS^36–38^. Another flavonoid kaempferol is a phytochemical commonly found in tea, spinach, kale, broccoli, and beans which is well known to interact with estrogen receptor and modulate its function. There is literature available suggesting its antibacterial activities along with ability to inhibit biofilm formation in *S. aureus* and *P. aeruginosa*. Even the lecithin, chitosan nanoparticles comprising of kaempferol are reported to interfere with QS machinery^39–42^. Evidence suggests kaempferol to inhibit QS in *Chromobacterium violaceum* where the authors have shown a reduction in violacein production (QS-driven phenotypic trait) when the bacteria was treated with the extract of *Cassia alata* containing kaempferol^43^. 7-Hydroxyflavanone is another 3-ring containing flavonoid which shows antibacterial activity yet is not established as a QSA molecule^44,45^. Capsaicin is an alkaloid commonly found in chilli peppers which shares a high degree of similarity with AHLs and is well established as a QSA in Gram negative microbes such as *P. aeruginosa, P. fluorescens* and *E. coli*^32,46–49^*.* Moreover, its wide spectrum QSA properties is identified against Gram positive bacteria like *S. aureus* and even in eukaryote like *Candida albicans*^48,50^. Resveratrol has an interesting role in nature, it is produced by the plant upon invasion of bacteria and/or fungus where this molecule protects the plant by exhibiting antibacterial and antifungal activity^51^. This molecule belongs to the family of phenylpropanoids and is a derivative of stilbene which is anthelmintic and antimicrobial in nature. The same molecule is also found to be produced by the microbe *Photorhabdus luminescens*, a Gram negative bacteria, seems to produce this molecule for surviving during competition with other molecules^52,53^. Moreover, in last five years, lot of research is done to explore the inhibitory effects of this compound on QS where it was found that, at effective concentrations below MIC levels significantly decreased the production of EPS and biofilm along with down regulation of Idh, relA, gtfC, and comDE genes in a human oral pathogen *Streptococcus mutans*. Also, the ability of the bacterium to utilize sugars and produce detrimental acid for oral health was found to be reduced upon treatment of resveratrol^54^. Further, there is strong evidence to suggest that this molecule is also affecting QS-driven EPS and biofilm production in *P. aeruginosa*^55^. A similar study to ours was also performed with resveratrol by Zhou et al. where they have proved resveratrol to inhibit QS, and increase susceptibility to antibiotic when P. aeruginosa was subject to the combinational treatment of aminoglycoside class of antibiotics and resveratrol^56^. Caffeic acid is a medicinal phenolic compound which is produced by a variety of consumable plant extracts such as coffee, tea, wine, and propolis. It is known to be a strong antimicrobial agent over and above it being antioxidant, anti-inflammatory and anticarcinogenic. In *Vibrio fischeri*, this compound is also reported to inhibit QS which was proven by reduction in its bioluminescence^57^. Ferulic acid, a hydroxycinnamic acid, is abundantly found in plant cell wall and is well used in Chinese medicine while, it is also known as antibacterial^58–60^. There is a paucity in data pertaining to antibacterial and QSA activity of batatifolin where Al-snafi showed its antibacterial activity^61^, while its QSA property is not being reported. Ajoene is a sulfur containing phytochemical isolated from garlic, providing a striking flavour and fragrance to food. This compound is well studied for its QSA trait where reports suggest its efficacy against QS in *P. aeruginosa*^62,63^. Moreover, this compound is also reported to have strong antibacterial activity against *P. aeruginosa* and *Campylobacter jejuni*^64,65^.

Our top hits consist of 6-Gingerol and Curcumin where, 6-Gingerol is a constituent of ginger and is a well-known QSA molecule. In our previous study we have shown it to suppress *V. fischeri* to suppress LuxR driven QS and we have previously reported this molecule to specifically suppress LasR mediated QS in *P. aeruginosa* to reduce biofilm formation^4,5,66^. Other constituents of ginger (*Zingiber officinale*), zingerone and shogaol also have varying degree of QSA properties^7^. Ginger is well known as traditional Indian spice and has several medicinal properties owing to its antibacterial and antifungal characters. The striking significance for 6-Gingerol to serve as QSA is that it resembles the structure of AHL and is sought to compete with the AHL molecules for acquiring active site on AHLr^5,7^. On the other hand, Curcumin is a Curcuminoid found in *Curcuma longa* (turmeric) a medicinal plant belonging to the ginger family. It is perceived as a wonder-phytochemical as it has anti- Alzheimer, cancer, apoptotic, inflammatory and oxidant properties. Apart from this, Curcumin is well known for its antibacterial and antifungal property for which it is widely used in traditional medicinal practices in India, China, and in other south-Asian countries. There are plentiful reports suggesting this wonder phytochemical to even serve as QSA which has been proved in *P. aeruginosa*, *C. albicans*, *Aeromonas hydrophila*^4,67–69^.

There are evidences where QSA molecules supress QS thereby allowing the increase in the susceptibility of bacteria to the antibiotic where experiments have shown that providing the QSA at sub-MIC levels in the culture along with antibiotic, shows rapid and steep inhibition in bacterial growth when compared to the levels of growth seen in absence of QSA and equal concentrations of the antibiotic ciprofloxacin, tetracycline, erythromycin and carbenicillin by using compound 14 as a lead phytochemical^70^. Similarly, in the current study we are also reporting 6-Gingerol and Curcumin to curb QS in *P. aeruginosa* while their supplementation with antibiotic ciprofloxacin at sub-MIC concentration improved antibiotic susceptibility thus corroborating the claims of Kim et al.^7^ and Guo et al.^70^.

In rundown, it very well may be inferred that the unconstrained restricting of 6-Gingerol and Curcumin with different AHLr of *P. aeruginosa* restrains different QS pathways thereby debilitating it ordnance, making it more susceptible to the antibiotics.
